# Spatial variability in herbaceous plant phenology is mostly explained by variability in temperature but also by photoperiod and functional traits

**DOI:** 10.1007/s00484-024-02621-9

**Published:** 2024-01-29

**Authors:** Robert Rauschkolb, Solveig Franziska Bucher, Isabell Hensen, Antje Ahrends, Eduardo Fernández-Pascual, Katja Heubach, Desiree Jakubka, Borja Jiménez-Alfaro, Andreas König, Tomáš Koubek, Alexandra Kehl, Anzar A. Khuroo, Anja Lindstädter, Faizan Shafee, Tereza Mašková, Elena Platonova, Patrizia Panico, Carolin Plos, Richard Primack, Christoph Rosche, Manzoor A. Shah, Maria Sporbert, Albert-Dieter Stevens, Flavio Tarquini, Katja Tielbörger, Sabrina Träger, Vibekke Vange, Patrick Weigelt, Aletta Bonn, Martin Freiberg, Barbara Knickmann, Birgit Nordt, Christian Wirth, Christine Römermann

**Affiliations:** 1 German Centre for Integrative Biodiversity Research (iDiv) Halle-Jena-Leipzig, Leipzig, Germany; 2 Institute of Ecology and Evolution with Herbarium Haussknecht and Botanical Garden, Friedrich Schiller University Jena, Jena, Germany; 3 Institute of Biology/Geobotany and Botanical Garden, Martin Luther University Halle-Wittenberg, Halle (Saale), Germany; 4 Royal Botanic Garden Edinburgh, Edinburgh, UK; 5 Biodiversity Research Institute, IMIB (Univ.Oviedo-CSIC-Princ.Asturias), Mieres, Spain; 6 Palmengarten and Botanical Garden Frankfurt, Frankfurt am Main, Germany; 7 Department of Botany, Faculty of Science, Charles University, Prague, Czech Republic; 8 Institute of Evolution and Ecology, University of Tübingen, Tübingen, Germany; 9 Department of Botany, University of Kashmir, Srinagar, Jammu & Kashmir India; 10 Institute of Biochemistry and Biology, Department of Biodiversity Research/Systematic Botany with Botanical Garden, University of Potsdam, Potsdam, Germany; 11 Institute of Plant Sciences, Ecology and Conservation Biology, University of Regensburg, Regensburg, Germany; 12 Petrozavodsk, Republic of Karelia Russia; 13 Department of Environmental Biology, Sapienza University of Rome, Rome, Italy; 14 Biology Department, Boston University, Boston, MA USA; 15 Botanic Garden Berlin, Freie Universität Berlin, Berlin, Germany; 16 Ringve Botanical Garden, NTNU University Museum, Norwegian University of Science and Technology, Trondheim, Norway; 17 Biodiversity, Macroecology and Biogeography, University of Goettingen, Goettingen, Germany; 18 Centre of Biodiversity and Sustainable Land Use, University of Goettingen, Goettingen, Germany; 19 Campus Institute Data Science, University of Goettingen, Goettingen, Germany; 20 Department of Ecosystem Services, Helmholtz-Centre for Environmental Research—UFZ, Leipzig, Germany; 21 Institute of Biodiversity, Friedrich Schiller University Jena, Jena, Germany; 22 Systematic Botany and Functional Biodiversity, Life Science, Leipzig University, Leipzig, Germany; 23 Core Facility Botanical Garden, University Vienna, Vienna, Austria; 24 Max-Planck-Institute for Biogeochemistry, Jena, Germany

**Keywords:** Botanical garden, Climate change, Flowering onset, Functional traits, Spatial variability, PhenObs

## Abstract

Whereas temporal variability of plant phenology in response to climate change has already been well studied, the spatial variability of phenology is not well understood. Given that phenological shifts may affect biotic interactions, there is a need to investigate how the variability in environmental factors relates to the spatial variability in herbaceous species’ phenology by at the same time considering their functional traits to predict their general and species-specific responses to future climate change. In this project, we analysed phenology records of 148 herbaceous species, which were observed for a single year by the PhenObs network in 15 botanical gardens. For each species, we characterised the spatial variability in six different phenological stages across gardens. We used boosted regression trees to link these variabilities in phenology to the variability in environmental parameters (temperature, latitude and local habitat conditions) as well as species traits (seed mass, vegetative height, specific leaf area and temporal niche) hypothesised to be related to phenology variability. We found that spatial variability in the phenology of herbaceous species was mainly driven by the variability in temperature but also photoperiod was an important driving factor for some phenological stages. In addition, we found that early-flowering and less competitive species characterised by small specific leaf area and vegetative height were more variable in their phenology. Our findings contribute to the field of phenology by showing that besides temperature, photoperiod and functional traits are important to be included when spatial variability of herbaceous species is investigated.

**Supplementary Information:**

The online version contains supplementary material available at 10.1007/s00484-024-02621-9.

## Introduction

During the past century, biomes around the world have experienced rapid changes in environmental conditions associated with increasing human activity (Jump and Peñuelas 2005; Steffen et al. 2015; Waters et al. 2016). Anthropogenic climate change has particularly intensified over the last several decades, resulting in higher temperatures (IPCC 2021), changes in precipitation patterns (Semmler and Jacob 2004; Dore 2005) and increased variability in environmental conditions (Gherardi and Sala 2019; IPCC 2021). In response to these changes, in particular to the rising temperatures, plants and animals have shifted their phenology, as well as patterns of abundance and distribution (Menzel and Fabian 1999; Parmesan and Yohe 2003; Menzel et al. 2006; Büntgen et al. 1968). Besides temperature (Parmesan and Yohe 2003; Menzel et al. 2006; Büntgen et al. 2022), precipitation (Craine et al. 2012; Shen et al. 2015) and photoperiod/sunshine hours (Basler and Körner 2012; Petterle et al. 2013; Rice et al. 2021; Ren et al. 2022) are considered important drivers of plant phenology. Regarding the increasing variability in climatic conditions, up to now, research has mainly focused on the impact of temporal variations on plant phenology. For example, remote sensing studies of deciduous forests and crops found that an increase in temperature variability is associated with an increase in the variability in the start and length of the growing season (Melaas et al. 2013; Zhang et al. 2014; Liu and Zhang 2020). In subalpine meadows in Colorado, interannual variability in flowering of herbaceous species was mainly determined by interannual variability in temperature and associated variability in snowmelt (Wadgymar et al. 2018). In contrast, the influences of spatial variability on phenology are less studied, but it is usually assumed that the correlations between the temporal variability in environmental parameters and variability in phenology would be the same as those for spatial variability (Peaucelle et al. 2019). However, phenological models are often not able to reproduce observed spatial variation (Richardson et al. 2011; Migliavacca et al. 2012) when applied at regional scales. For example, shifts in the flowering onset of wildflowers as a response to changing temperatures in spring are strongly dependent on spatial locations (Willems et al. 2021; Lee et al. 2022). As spatial variability in phenology may affect the magnitude of biotic interaction between plants (e.g. facilitation and competition, Yang and Rudolf 2010), and between plants and animals (e.g. pollination and herbivory, Yang and Rudolf 2010; Forrest 2015; Inouye 2022), it is important to develop a profound understanding of the drivers (Stemkovski et al. 2023), especially since the main interest in these areas has so far been on analysing means and overlooking the importance of variability in space and time (Wetzel et al. 2023).

In this current study, we focus on the spatial variability of phenological events in perennial herbaceous species. Although herbaceous species represent > 85% of species found in temperate ecosystems (Ellenberg et al. 2010), they are still underrepresented in phenological studies as compared to trees, shrubs and crops (Clarivate Web of Science search conducted on 16th February 2023 using the following terms: Phenolog* AND herbaceous/herbs/herb/grass/grasses = 2774 papers, Phenolog* AND tree*/shrub* = 9945 papers, Phenolog* AND crop* = 9412 papers). We assume that the variability of the abiotic factors mentioned above (temperature, precipitation and photoperiod) will be key driving factors. In addition, species-specific and growth form-specific phenological responses to climatic conditions in herbaceous species can also be described by using functional traits (Sun and Frelich 2011; König et al. 2018; Bucher et al. 2018; Bucher and Römermann 2021; Sporbert et al. 2022; Horbach et al. 2023). Most of these studies, however, focus on the onset of a phenological stage and linear shifts in response to temperature rather than the variability in phenological stages, e.g. differences in the timing of phenological events over several years or across sites, but see Osada (2020) for an example on the variability in phenological stages in trees and Stemkovski et al. (2023) for an example across woody species, grasses and herbaceous species. In an effort to contribute to a profound understanding of spatial variability in the phenology of herbaceous species, we investigate the relative impact of the variability in environmental conditions and plant functional traits on the variability in vegetative and reproductive phenology in herbaceous plant species.

More specifically, we quantified the spatial variability in phenology using large-scale phenology data collected mainly in 2022 from 148 perennial herbaceous species cultivated in 15 botanical gardens included in the PhenObs network (www.idiv.de/en/phenobs.html). PhenObs researchers follow standardised protocols to monitor the phenology of perennial herbaceous species in botanical gardens (Nordt et al. 2021). The resulting phenological records capture the whole life-cycle of the studied species across the year including the onset, end and duration of vegetative (i.e. initial leaf growth and senescence) and reproductive (i.e. flowering and fruiting) phenological stages; most other studies focus on just one phenological phase (for example, only flowering) or even just part of one phase (the start of flowering).

We focused on four continuous traits—temporal phenological niche, seed mass, vegetative height and specific leaf area (SLA)—which are described as core functional traits of plants (Weiher et al. 1999) and the latter being part of the LHS plant ecology strategy scheme proposed by Westoby (1998). Regarding the phenological niche, previous studies have shown that early-flowering species generally show a stronger response to increases in temperature than later-flowering species (Fitter and Fitter 2002; Dunne et al. 2003; Miller-Rushing et al. 2007; König et al. 2018; Renner and Zohner 2018), and accordingly, we expect that the phenology of early-flowering species is more variable than that of late-flowering species in response to climate variability. Small-seeded species tend to follow ruderal strategies, have a wider overall geographic distribution, often grow under more variable environmental conditions (Tautenhahn et al. 2008; Thomson et al. 2011) and are therefore predicted to exhibit greater variability in phenology (Sultan 2001; Richards et al. 2005; Sides et al. 2014; Fajardo and Siefert 2019). More competitive species indicated by larger height and higher SLA are predicted to be less variable in their phenology than smaller species with lower SLA (Gaudet and Keddy 1988; Moles et al. 2009; König et al. 2018).

In this study, we aim to answer the following questions:

1. To what extent do herbaceous species observed in botanical gardens differ in their spatial variability in vegetative and reproductive phenology?
2. Which environmental factors and functional traits are the most important to predict spatial variability in the phenology of herbaceous species?

Finally, we assess the ecological relevance of spatial variability of phenological events in herbaceous species in association with climate change.

## Material and methods

### Phenological data

We analysed phenological records of 148 perennial herbaceous plant species from 54 plant families, which were monitored in 15 botanical gardens in the framework of the PhenObs network (Nordt et al. 2021, www.idiv.de/en/phenobs.html). Plants in 13 gardens were monitored in 2022. For two gardens, we included data collected in 2020 (Edinburgh) and 2021 (Rome), as these gardens did not monitor plants in 2022 (see Table 1 for the geographic location of the 15 gardens and Table ESM 2.1 for a list of species included in this study). At least, observations of three different gardens were available for each of the selected species. The number of different botanical gardens for each species varied between three and 13, since not all species were observed in every garden. We standardised the names of species using ‘The Leipzig catalogue of vascular plants’ (LCVP; Freiberg et al. 2020).

**Table 1:** Overview of the botanical gardens including ISO country codes (see Fig. ESM 1.5 for a map) with the mean temperature (°C) in the period from January to December 2022, the geographic coordinates, the variability in photoperiod during growing season as the difference of the longest and shortest day and the number of species are included in the analyses

City of the botanical garden	Mean temperature °C	Latitude	Longitude	Variability in photoperiod hh:mm	Number of included species
Berlin (DE)	11.4	52.454	13.305	09:10	88
Edinburgh (UK)	8.5	55.965	−3.209	10:40	19
Frankfurt (Main) (DE)	11.8	50.123	8.656	08:21	68
Halle (Saale) (DE)	10.8	51.488	11.960	08:49	109
Jena (DE)	9.9	50.930	11.585	08:37	105
Leipzig (DE)	11.1	51.328	12.392	08:45	64
Petrozavodsk (RU)	4.2	61.768	34.401	14:24	18
Potsdam (DE)	11.4	52.404	13.025	09:09	77
Prague (CZ)	10.8	50.071	14.420	08:21	56
Rome (IT)	17.4	41.891	12.463	06:06	29
Srinagar (IN)	12.0	34.127	74.832	04:34	20
Trondheim (NO)	5.5	63.446	10.452	16:08	54
Tuebingen (DE)	11.8	48.539	9.035	07:51	52
Vienna (AT)	11.9	48.192	16.380	07:44	76
Xixón (ES)	15.8	43.520	−5.614	06:29	22

PhenObs members monitored the phenology of marked accessions of the listed plant species at their botanical gardens following the standard protocol described in Nordt et al. (2021). As typically, the number of individuals (one to hundreds) or the area covered (single plants to several m^2^) per accession varies strongly between species, we collected the data in a standardised way and observed an approximately 1 m^2^ plot per species in each garden. Though the exact number of individuals per accession is not known, the density per species within the plot is comparable across the gardens. The monitoring was done on a weekly basis. This interval is often recommended in order to obtain the most accurate resolution of the phenological events at the accession level with a feasible amount of time (Miller-Rushing et al. 2008; Cornelius et al. 2011; Nordt et al. 2021). From these data, we extracted annual records for the day of the year (DOY) of the two vegetative (leaf unfolding and onset of leaf senescence at the end of the growing season) and three reproductive (onset of flowering, peak flowering and onset of fruiting) phenological stages. In addition, flowering duration was defined as the period in days between the first and last day of flowering, where flowering duration of species that were observed with open flowers only on one single weekly monitoring event was set to 1. In order not to make the data analysis too complex, and since the onset and end of flowering are strongly correlated (Nordt et al. 2021), we therefore assume similar patterns for both phenological stages; we have not analysed the end of flowering in this paper. All stages are described in detail in Table 2. For analysing the species-specific differences in the spatial variability in phenological stages, we calculated the standard deviation (SD_Phen_) across gardens for each species. Since events such as start of leaf senescence that occurred at the end of December in 1 year in one garden and the beginning of January in another year in a different garden would lead to disproportionately large standard deviations, we standardised (std. DOY) the measures of the day of the year (DOY) by considering their distance to the centre of a year (1^st^ of July respectively DOY 182).

**Table 2:** Two vegetative and four generative phenological stages used in this study, following the PhenObs protocol (Nordt et al. 2021)

Phenological stage	Description
Leaf unfolding	Day of the first fully visible leaf
Onset senescence	Day when 5% of species’ leaves started to change their colour as a sign of losing chlorophyll, dried out or fell off but excluding temporal responses to drought or herbivory
Onset of flowering	Day of the first fully open flower
Peak flowering	Day of the maximum number of open flowers (derived from flowering intensity)
Flowering duration	Period between the first day of flowering and the day when the last flower with visible anthers can be observed
Onset of fruiting	Day of the first ripe fruits

### Environmental data

Table 3 gives an overview on all environmental parameters included in this study. As there were no standardised measuring devices for temperature set-up in the gardens, we used gridded data from the Climate Research Unit (CRU, Camarillo-Naranjo et al. 2019; Harris et al. 2020) and extracted mean monthly temperatures of the 0.5° grid cell for each botanical garden. We calculated the mean temperature for the 4-month period before a phenological event occurred and calculated the standard deviation of temperature (SD_Temp_) per species across all gardens where the species was monitored. We did not include precipitation in our analyses, as plants were watered in most of the botanical gardens during periods of dry weather. We included the latitude of the botanical gardens in which a species was monitored to capture the spatial variability in photoperiod and seasonality and calculated the corresponding standard deviation thereof (SD_Lat_) per species.

**Table 3:** Environmental factors and functional traits used in this study including information about their origin and the reported links to the variability in phenological events. The number of species for which a functional trait was available is shown as *n*

Description	Source	Reported link to variability in phenological stages
Environmental factors		
Variability in temperature (SD_Temp_)	Mean temperature for the 4-month period before a phenological event occurred (respectively end of flowering for the flowering duration); standard deviation per species across gardens for which phenological data of the species was included	Greater variability in a more heterogeneous environment (Wadgymar et al. 2018; Liu and Zhang 2020)
Variability in latitude factors (SD_Lat_)	Calculation of the standard deviation of the latitudinal distance between the gardens in which the species was monitored	Greater variability in a more heterogeneous environment (Wadgymar et al. 2018; Liu and Zhang 2020)
Variability in local conditions in the different botanical gardens (#Gardens)	Number of botanical gardens in which monitoring was conducted for this species	Greater variability in a more heterogeneous environment (Wadgymar et al. 2018; Liu and Zhang 2020)
Functional traits		
Mean day of flowering onset [DOY]; *n* = 147	Extracted for each species from the PhenObs data used in this study	Greater variability in early-flowering species (Fitter and Fitter 2002; Dunne et al. 2003; Miller-Rushing et al. 2007; Renner and Zohner 2018)
Vegetative height [m]; *n* = 122	Extracted means from the TRY database (Kattge et al. 2020) supplemented with measurements from the PhenObs network (Nordt et al. 2021)	Increased competitive ability of taller species, which should be less variable in their phenology (Gaudet and Keddy, 1988; Moles et al. 2009; König et al. 2018)
Mass for an individual seed [mg]; *n* = 129	Extracted means from the TRY database (Kattge et al. 2020) supplemented with measurements from the PhenObs network (Nordt et al. 2021)	Presumed wider distributional range in species with smaller seeds (Thomson et al. 2011) and therefore a greater variability (Sultan 2001; Richards et al. 2005; Sides et al. 2014; Fajardo and Siefert 2019)
Specific-leaf-area (SLA) [mm^2^/mg]; *n* = 124	Extracted means from the TRY database (Kattge et al. 2020) supplemented with measurements from the PhenObs network (Nordt et al. 2021)	Increased competitive ability of species with high SLA species, which should be less variable in their phenology (Gaudet and Keddy, 1988; Moles et al. 2009; König et al. 2018)

In general, we assume that plants grow under favourable conditions in botanical gardens, especially with regard to water (Primack and Miller-Rushing 2009). Nonetheless, the influence of maintenance factors such as shading, irrigation frequency and weeding schedule, as well as soil type and microclimate conditions, can differ significantly among gardens. Because it is difficult to quantify the possible variability caused by these factors (Sporbert et al. 2022), we added the number of gardens in which the phenology of a species was observed as an additional explanatory variable under the assumption that the inclusion of more gardens should increase the variability in these factors.

### Plant functional traits

We included four functional traits in this study—temporal phenological niche, seed mass, vegetative height and SLA, as prior research by ourselves and others had shown that these nicely capture plant ecology strategies (leaf-height-seed (LHS) plant ecology strategy scheme, Westoby 1998) and have particular importance for phenology (see Table 3 for referenced reasoning and introduction). In addition, all traits are widely available in databases (e.g. TRY Kattge et al. 2020) or easy to measure. In order to be able to better depict the variability between species, we decided to use traits measured on a continuous scale and not to use categorical traits (Funk et al. 2017).

We calculated the species-specific trait ‘temporal niche’ from the phenological data by calculating the mean of the day of first flowering across all gardens and years. For vegetative height, seed mass and SLA, we extracted data from the TRY database (Kattge et al. 2020) and calculated the mean of all entries available for the respective species. We filled the gaps within the data set (c. 10% of all cells in the trait matrix i.e. 39 of 375 trait values) with measurements conducted by PhenObs members as part of the PhenObs protocol (Nordt et al. 2021; see Sporbert et al. 2022 for a detailed description of the methods used). We assume that differences among the studied species for these traits are far greater than any differences that would result between the two types of data source, and therefore bias associated with sources of data is not expected. Linking seed mass with the distributional range size of a subset of the studied species for which data was available in the Global Inventory of Floras and Traits—GIFT (Weigelt et al. 2020) confirmed our assumption that small-seeded species have a wider global distribution as we found a significant negative association (Pearson’s r = −0.32, *n*_Species_ = 100, *p* < 0.01). See Table ESM 2.2 for a species-specific overview of the functional traits and the corresponding distributional range size extracted from GIFT.

### Data analysis

#### Differences in the spatial variability in phenology

To test whether the spatial variability in vegetative and reproductive phenology of herbaceous species observed in botanical gardens differ, we performed Levene’s tests using the R-package *rstatix* (Kassambara 2023). Here, observed DOYs of the different phenological stages were included as dependent; the botanical gardens were included as explanatory variables. Levene’s test is usually used to analyse whether data sets have a comparable variance and is therefore a suitable statistical method to test for differences in the spatial variability as represented by the observations in different botanical gardens.

#### Relative importance of traits and environmental factors for the variability in phenology

First, for reasons of data exploration and support for the discussion, we performed a principal component analysis (PCA) using the *prcomp* function from base R on the species x traits matrix to investigate the overall patterns and potential associations within the four selected traits’ phenological niche, seed mass, plant height and SLA. As the species traits matrix was not complete, and as PCA cannot cope with missing data, only 118 out of 148 species were included in this pre-analysis.

To analyse the relative importance of SD_Temp_, SD_Lat_, the number of gardens and functional traits (see Table 3) for explaining the species-specific differences in the spatial variability in phenological stages, we used boosted regression trees (BRTs, Elith et al. 2008). This approach has the great advantage of allowing large data sets with numerous different predictor variables to be evaluated (see also Sporbert et al. 2022). Furthermore, this method is relatively insensitive to collinearity, missing values in the predictor variables are handled with minimal loss of information by using surrogates, and it accounts for non-linear effects (Elith et al. 2008; Bianchini and Morrissey 2020; Cai et al. 2023).

Phenological patterns often show phylogenetic non-independence, and such effects may be even greater in botanical gardens where plants are growing under favourable conditions (Primack and Miller-Rushing 2009; Yang et al. 2021). Therefore, we included phylogenetic eigenvector regressions in our analyses (Diniz-Filho et al. 1998; Bianchini and Morrissey 2020; Sporbert et al. 2022). These eigenvectors can control for phylogenetic autocorrelation in BRTs, when a sufficiently high number of eigenvectors, representing approximately 90% of the phylogenetic structure, are included. We created a phylogenetic tree of the species using the function *phylo.maker* from the R-package *V. Phylomaker* 0.1.0 (Jin and Qian 2019). From this tree, we calculated a pairwise distance matrix and extracted the eigenvectors using a principal coordinate analysis (PCoA) provided by the *pcoa* function from the *ape* package version 5.6.-1 (Paradis and Schliep 2019). To represent 90% of the phylogenetic structure of the distance matrix, we included the first 39 of the total 147 eigenvectors in our BRT models (Table ESM 2.3).

In order to achieve a normal distribution of the data, we ln-transformed the variabilities in the phenological stage and the corresponding temperature, as well as the SLA, vegetative height and seed mass in prior to the analyses. We fitted the different BRT models using the *gbm.step* function from the *gbm* package version 2.1.8 (Greenwell et al. 2022), with a Gaussian error distribution and a fraction of training data of 0.5, a learning rate of 0.01, a tree complexity of 1 and a tolerance of 0.01. After running the model once, we simplified them by dropping predictors using the *gbm.simplify* function from the R-package *dismo* (Hijmans et al. 2022). Finally, we used cross-validation correlations to assess the performance of our final models. All statistical analyses were done in R 4.1.0. (R Core Team 2022).

## Results

**Fig. 1:**
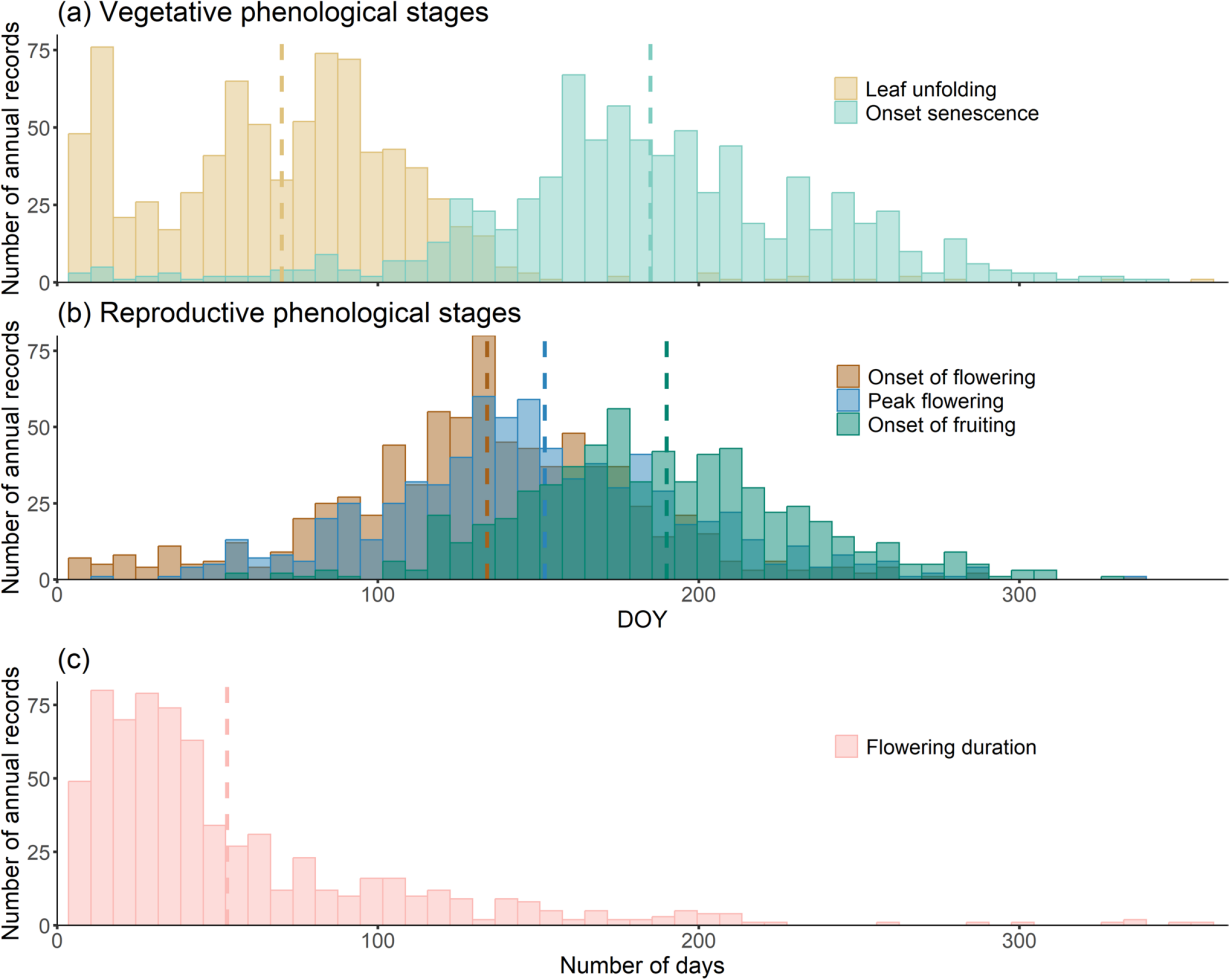
Overview of the six phenological stages including the number records with a certain day of the year (DOY) and a number of days for flowering duration, respectively. Only data points of species observed in three or more botanical gardens are considered; **a** shows the day of the year (DOY) of the vegetative stages onset of leaf unfolding (*n*_Records_ = 812, *n*_Species_ = 148) and senescence (*n*_Records_ = 783, *n*_Species_ = 148), **b** shows the day of the year (DOY) of the reproductive stages onset of flowering (*n*_Records_ = 784, *n*_Species_ = 147), peak flowering (*n*_Records_ = 737, *n*_Species_ = 147), onset of fruiting (*n*_Records_ = 638, *n*_Species_ = 145), **c** shows the duration of flowering (in days) (*n*_Records_ = 725, *n*_Species_ = 147). The dashed lines indicate the associated mean value across species, gardens and years

Across species and botanical gardens, average leaf unfolding occurred on DOY 70 +/− 28 (11th of March), followed by the onset of flowering on DOY 134 +/− 39 (14^th^ of May), the peak flowering on DOY 152 +/− 42 (1^st^ of June), the onset of leaf senescence on DOY 185 +/− 35 (4^th^ of July) and the onset of fruiting on DOY 190 +/− 38 (9^th^ of July) (Fig. 1a, b and see Table ESM 2.4 for species-specific values). Across all species, we found a mean flowering duration of 53 +/− 37 days (Fig. 1c). There were also single records across species and gardens that either showed these events particularly early or late in the year (Fig. 1). Similarly, flowering duration of species showed particularly short or long periods for certain species and gardens (Fig. 1c, range of flowering duration between 1 day in 37 annual records of 54 species and 357 days in one record of one species).

**Fig. 2:**
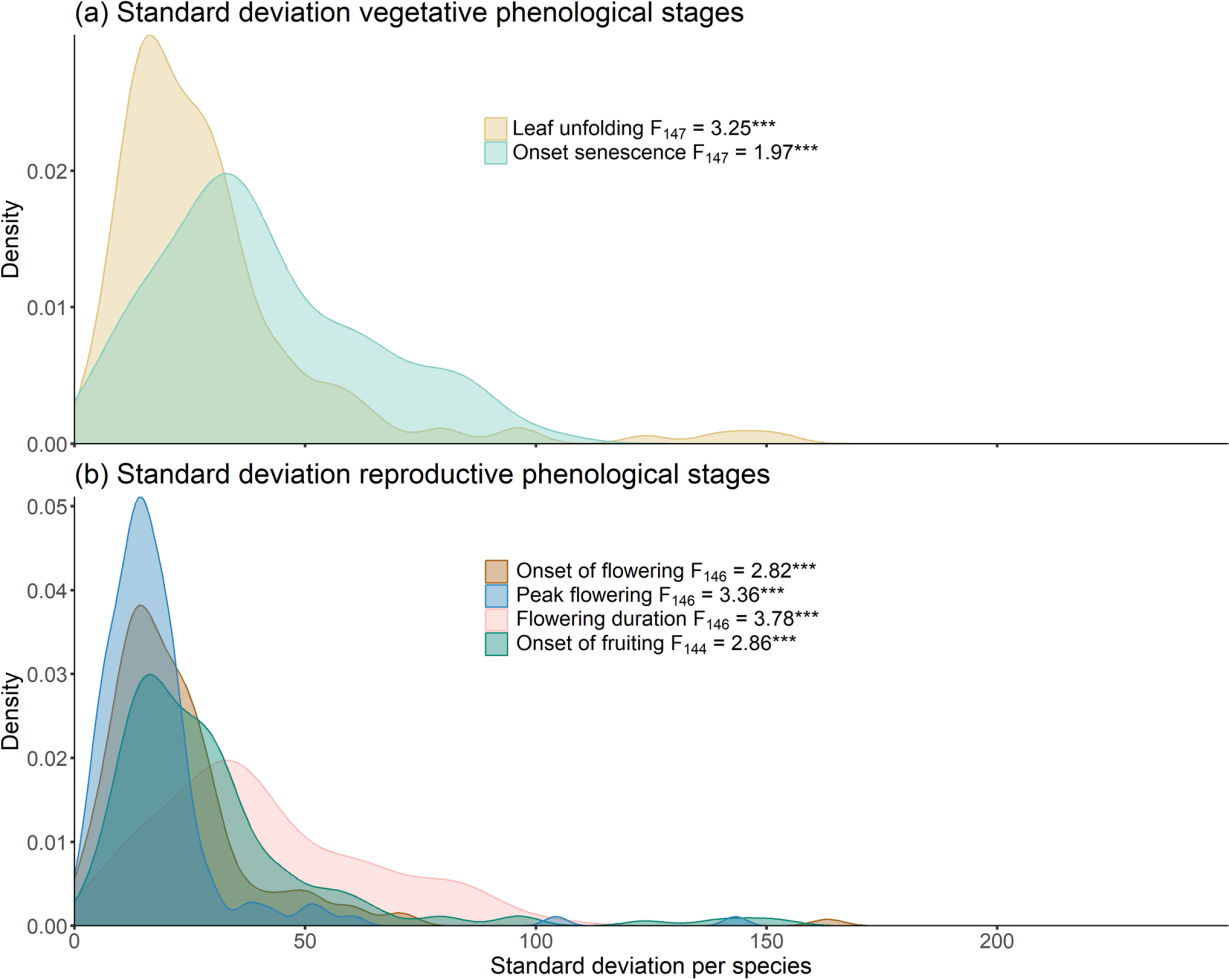
Proportion of the differently calculated standard deviations per species for the six phenological stages including Levene’s test statistics comparing the different species (*F*-value, degrees of freedom and significance (***p < 0.001); **a** shows the two vegetative stages and **b** the four reproductive stages

We found significant differences between the studied species in the spatial variability for all six phenological stages (Fig. 2; *p*-values of the Levene’s tests for all phenological stages < 0.001). Figure 2 shows that the variability for the onset of flowering and the peak flowering was the least different among the species, whereas for the variability in the onset of senescence and flowering duration differences were largest.

**Fig. 3:**
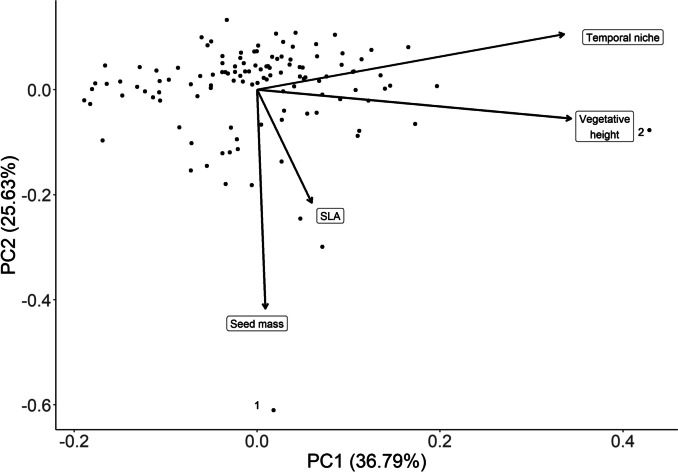
PCA biplot ran with a subset of all species (*n* = 118, each dot is one species), based on the four functional traits temporal niche (expressed by the mean DOY for the onset of flowering), seed mass, vegetative height and specific-leaf-area (SLA). Examples for species with notably high values are, for seed mass, ^1^*Paeonia officinalis* and for vegetative height, ^2^*Humulus lupulus*

The PCA did not reveal any grouping of the functional traits. However, some species at the edges of the main cloud of data points were determined by notably high values for particular traits, such as vegetative height (e.g. *Humulus lupulus* with 300 cm, which is a climbing plant with an especially large vegetative height) and seed mass (e.g. *Paeonia officinalis* with 106 mg) (Fig. 3). PC1 was mainly associated with temporal niche (mean day of flowering onset) and vegetative height, while PC2 was mainly associated with seed mass. SLA was most important for PC3 (not shown in Fig. 3; see Table ESM 2.5). Furthermore, the results suggest that in this multivariate approach, vegetative height was positively correlated with the temporal niche indicating that taller growing species flower later.

**Fig. 4:**
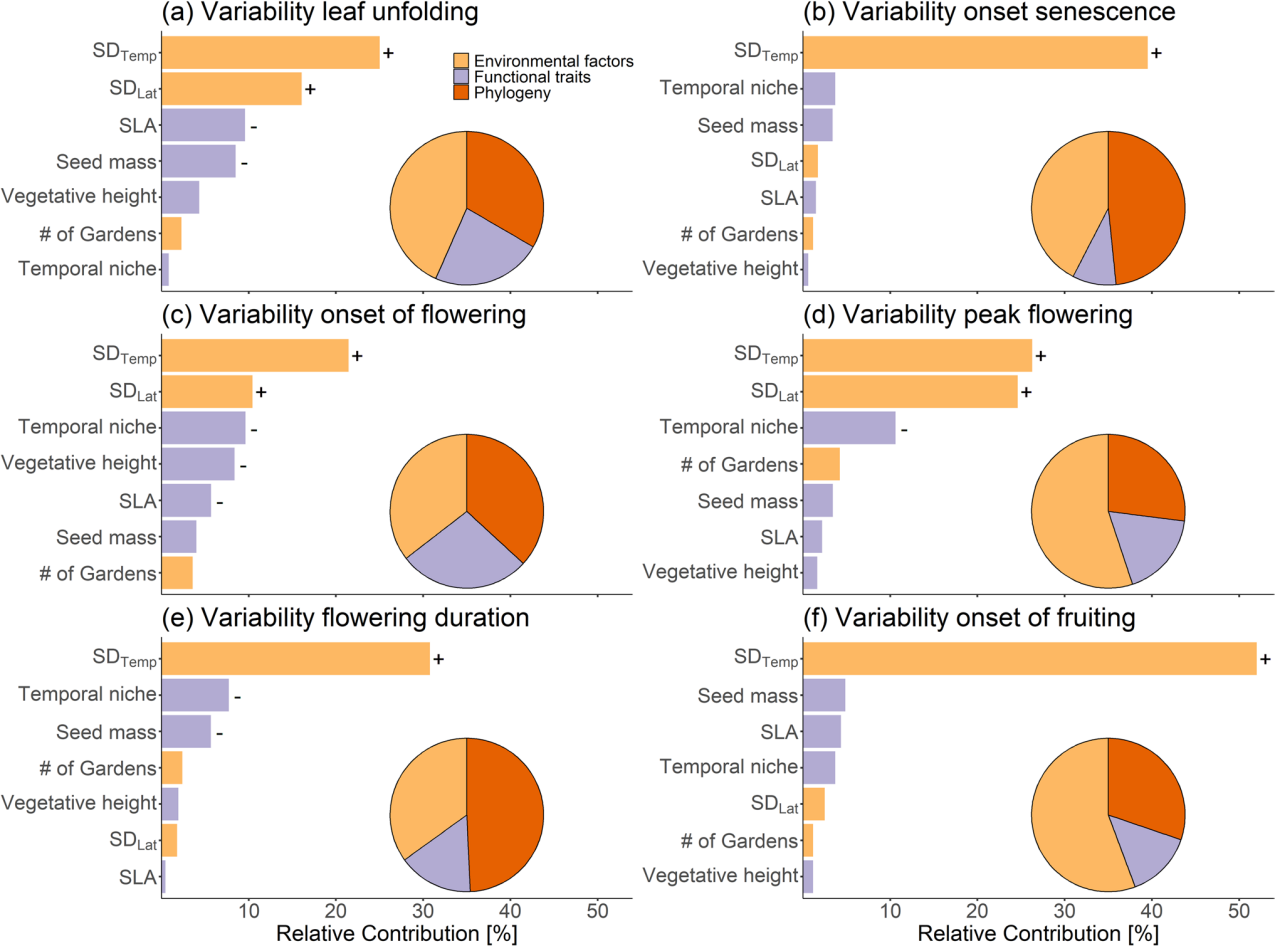
Relative importance (%) of the variability in environmental factors and species’ functional traits on the variability of six phenological stages, deduced from boosted regression trees (BRTs), in which 49 phylogenetic (Table ESM 2.2) eigenvectors were included. Pie charts represent the summed-up contributions of the variables grouped by ‘Environmental factors’, ‘Functional traits’ and ‘Phylogeny’. Boosted regression tree models were fitted for **a** variability in leaf unfolding (*n* = 148, cross-validation correlation *R*^2^ = 0.42), **b** variability in leaf senescence (*n* = 148, *R*^2^= 0.43), **c** variability in the onset of flowering (day of year; *n* = 147, *R*^2^ = 0.44, **d** variability in the peak flowering (day of year; *n* = 146.7 *R*^2^ = 0.49), **e** variability in the flowering duration (day of year; *n* = 145, *R*^2^ = 0.56) and **f** variability in the onset of fruiting (day of year; *n* = 145, *R*^2^ = 0.48). For relative contribution of > 5%, the direction of the association is indicated by the sign next to the bar (‘+’ = positive association, ‘−’ = negative association)

Overall, the most parsimonious BRT models of the effects of environmental factors and species’ functional traits on the spatial variability in phenological stages showed moderate to high levels of cross-validation correlation (*R*^2^ = 0.42–0.56), indicating good accuracies. We found strong positive associations between the variability in temperature (SD_Temp_) and the variability of all studied phenological stages. For all cases, SD_Temp_ contributed more than 25% to explain the spatial variability in phenology. In contrast, the variability in latitude (SD_Lat_) was only important for explaining the variability in leaf unfolding, flowering onset and flowering peak. The variability in local conditions as represented by the numbers of gardens where the species were monitored was negligible for all phenological stages. In all cases, the variability of these environmental factors had a positive impact on the variability in phenology (Fig. 4; see Fig. ESM 1.6 for partial dependency plots).

With regard to the functional traits, the results of the BRTs showed that competitive species (high SLA and/or taller growth) were less variable in leaf unfolding and flowering onset (Fig. 4a, c; Fig. ESM 1.6 for partial dependency plot), and species with smaller seeds were less variable in leaf unfolding and flowering duration (Fig. 4a, e; Fig. ESM 1.6 for partial dependency plots). Early-flowering species showed a more variable timing for the display of first flowers, the peak flowering and the flowering duration (Fig. 4c, d, e; Fig. ESM 1.6 for partial dependency plots). In contrast, functional traits had only a marginal impact on the variability of the onset of senescence.

The phylogenetic signal, indicated by the sum of the included eigenvectors (see pie-charts in Fig. 4), was explained between 27% (SD peak flowering) and 49% (SD flowering duration) of overall variation, but each single eigenvector did not explain more than 9% (Fig. ESM 1.6).

## Discussion

Our results showed clearly that herbaceous species in botanical gardens differ in their spatial variability in vegetative and reproductive phenological stages. We found that the spatial variability in temperature resulting from the distribution of botanical gardens is the most important factor driving the spatial variability in herbaceous species’ phenology. In addition, spatial variability in photoperiod also has an important influence on the variability in phenology, but this depended on the investigated phenological stage. The observation that increased spatial variability in temperature led to an increase in the variability in phenological stages is consistent with our predictions and previous studies (Melaas et al. 2013; Zhang et al. 2014; Wadgymar et al. 2018; Liu and Zhang 2020). The largest proportion of observed species was native to the regions of the botanical gardens or at least can survive frosts in winter without major protective efforts as they originate from comparable climatic zones (e.g. *Helleborus orientalis* (Turkey), *Podophyllum peltatum* (North America) and *Trillium sessile* (North America)). Therefore, we assume that the strong impact of temperature was not due to the observations of species that were not growing under their optimal temperature conditions and related stronger responses to variability in temperature as proposed by Defila and Clot (2001) and Larcher (2010).

So far, numerous studies have shown that photoperiod strongly influences tree phenology (Hunter and Lechowicz 1992; Basler and Körner 2012; Petterle et al. 2013; Peaucelle et al. 2019). In contrast, this influence on herbaceous species has been assumed but hardly tested by data (Ahmad et al. 2021; Rice et al. 2021; Ren et al. 2022). Here we could show that greater variability in photoperiod positively relates to greater variability in leaf unfolding, flowering onset and flowering peak. Regarding leaf unfolding, future physiological studies are needed to understand the mechanisms behind, as many perennial herbaceous species usually overwinter below the leaf litter or deeper in the soil where light may be blocked (Raunkiaer 1934; Facelli and Pickett 1991). It is known that provenance, indicated by adaptations to local conditions, is crucial for phenological patterns in herbaceous species (Gugger et al. 2015; Rauschkolb et al. 2023). Precise information about the genetic origin of the accessions are typically not available for the species grown in botanical gardens. Therefore, we cannot separate the effects of genetic diversity of the monitored accessions in different gardens from the variability of environmental factors to explain the spatial variability in phenology. However, if we assume that genetic variation increases with the number of gardens where a species was observed, our results would indicate a small impact of genetic variation (Fig. 4). Due to missing information (e.g. origin, number of cultivated generations, age of the monitored individuals), it is also not possible to determine whether the observed phenological patterns are locally adapted or represent plastic responses. To address such questions, common garden experiments with identical genetic origins have to be established (e.g. see Renner and Chmielewski 2022 for an example of trees in botanical gardens).

For some phenological stages, we could confirm our assumptions that early-flowering and/or less competitive species that were smaller are more variable. The observation that early-flowering species are more variable in their phenology (higher variability in the onset and peak flowering, as well as in flowering duration) coincides with many other studies (Fitter and Fitter 2002; Dunne et al. 2003; Miller-Rushing et al. 2007; Renner and Zohner 2018; Stemkovski et al. 2023) and may be explained by their generally enhanced response to changes in the environments.

The detected negative association between vegetative height and SLA with the variability in leaf unfolding and in the onset of flowering (Fig. 4a, d) was expected, as less competitive species (indicated by smaller height and SLA) avoid competition by showing stronger phenological plasticity (König et al. 2018). The same applies to the negative association found between seed mass and the variability in leaf unfolding and flowering duration. In summary, particularly for spatial variability in early stages like leaf unfolding and flowering onset, we showed that especially the traits from the LHS strategy scheme explained a relatively large proportion of the observed phenological patterns. This is remarkable as specifically these earlier stages were shown to be less variable when comparing species, suggesting that trait differences between species matter less. Here, we may speculate that later phenological stages, such as fruit ripening or senescence, are more strongly influenced by other factors than traits in the botanical gardens (e.g. pruning, irrigation regimes, collection of flowers and fruits by gardeners and visitors). However, assuming that the number of gardens per species proxies the variability in these factors, this hypothesis cannot be confirmed by our analyses, and further studies may need to investigate the impact of these management treatments being specific to botanical gardens.

Our results suggest that the spatial variability in phenology, like temporal variability (Zhang et al. 2014; Liu and Zhang 2020), is mainly driven by the spatial variability in temperature. However, when the phenology of herbaceous species is modelled on spatial scales, further factors, depending on the investigated phenological stage, should be considered. This includes photoperiod and species-specific responses to environmental conditions. Here, we found that these species-specific responses can be efficiently and precisely represented by continuously measurable functional traits.

The observation that the spatial variability in phenology of herbaceous species is not only driven by the variability in temperature may explain why phenological events do not keep pace with climate change as suggested previously (Huang et al. 2017; Peaucelle et al. 2019). The ability to phenologically adapt to changes in climate is considered to be crucial to reduce the risk of local extinction (Dawson et al. 2011). Furthermore, as for onset of flowering and flowering peak, we found a significant high impact of photoperiod and functional traits on the spatial variability in phenology. We conclude that plant-pollinator interactions (Yang and Rudolf 2010; Inouye 2022) may, in particular, be influenced by herbaceous plant species losing pace with climate change, leading to biotic mismatches.

Although the observations for this study were made in botanical gardens, which may differ in local conditions (e.g. maintenance, soil texture, irrigation), the variability in these factors (measured by the number of gardens where a species was observed) had only a negligible impact on the variability in phenological stages. Therefore, we conclude that the botanical gardens participating in the PhenObs network indeed provide a sufficiently controlled platform for phenological research (Primack and Miller-Rushing 2009; Sporbert et al. 2022). Regarding possible, even stronger associations between functional traits and phenological patterns, as observed in other studies in botanical gardens (Sporbert et al. 2022; Horbach et al. 2023), we consider two reasons which may have obscured our analyses. First, the observed populations have different genetic origins at each garden and therefore may show different responses to environmental factors. As a result of this genetic variation, they can also differ greatly in their phenotypic plasticity, which may lead to divergent patterns of variability in phenology (Matesanz and Ramíres-Valiente 2019). Secondly, as data on functional traits measured directly in the botanical gardens were not yet fully available, we were not able to use such species- and garden-specific trait data for our analyses, even though these may also impact the analyses as changes in phenology may go along with changes in functional traits measured on the same population (see e.g. Bucher et al. 2018). Adding additional traits from the trait spectrum, such as belowground traits, may complement the analyses. However, these traits are not available for most of the species. In addition, we have selected SLA, vegetative height and seed mass as these were shown to represent a broad spectrum of ecological strategies in plants (Westoby 1998).

To gain an even more profound understanding of the spatial variability in the phenology of herbaceous species, we suggest for future studies to include aspects of intraspecific trait variability and environmental parameters measured in each garden (e.g. light availability, microclimate, soil type, watering regimes, weeding frequency, etc.). This would provide a better connection among phenological traits measured at botanical gardens and current environmental conditions in the natural environment and thus reveal how future plants growing at botanical gardens and in the wild will be affected by and cope with climate change.

### Supplementary information

ESM 1 (DOCX 1895 kb)

ESM 2 (XLSX 52 kb)

## Data Availability

The data that support the findings of this study are openly available in the ‘iDiv Data Repository’ at 10.25829/idiv.3535-6j8cmx, 10.25829/idiv.3536-o94ra8 and 10.25829/idiv.3550-m3qf86.
