# Supplementary material for: Spatial variability in herbaceous plant phenology is mostly explained by variability in temperature but also by photoperiod and functional traits
Source: Int J Biometeorol. 2024 Jan 29;68(4):761–75. doi: 10.1007/s00484-024-02621-9 (PMC10963576; doi:10.1007/s00484-024-02621-9)
Supplement: Supplementary file 1 — (DOCX 1895 kb) [file 484_2024_2621_MOESM1_ESM.docx]

**Supplementary**

*Spatial variability in herbaceous plant phenology is mostly explained by variability in temperature but also by photoperiod and functional traits*

International Journal of Biometeorology

Robert Rauschkolb^1,2*^, Solveig Franziska Bucher, Isabell Hensen, Antje Ahrends, Eduardo Fernández-Pascual, Katja Heubach, Desiree Jakubka, Borja Jiménez-Alfaro, Andreas König, Tomáš Koubek, Alexandra Kehl, Anzar Khuroo, Anja Lindstädter, Faizan Shafee, Tereza Mašková, Elena Platonova, Patrizia Panico, Carolin Plos, Richard Primack, Christoph Rosche, Manzoor A. Shah, Maria Sporbert, Albert-Dieter Stevens, Flavio Tarquini, Katja Tielbörger, Sabrina Träger, Vibekke Vange, Patrick Weigelt, Aletta Bonn, Martin Freiberg, Barbara Knickmann, Birgit Nordt, Christian Wirth, Christine Römermann

*Corresponding author: Robert Rauschkolb, Robert.Rauschkolb@uni-jena.de

^1^ German Centre for Integrative Biodiversity Research (iDiv) Halle-Jena-Leipzig, Leipzig, Germany
^2^ Institute of Ecology and Evolution with Herbarium Haussknecht and Botanical Garden, Friedrich Schiller University Jena, Jena, Germany


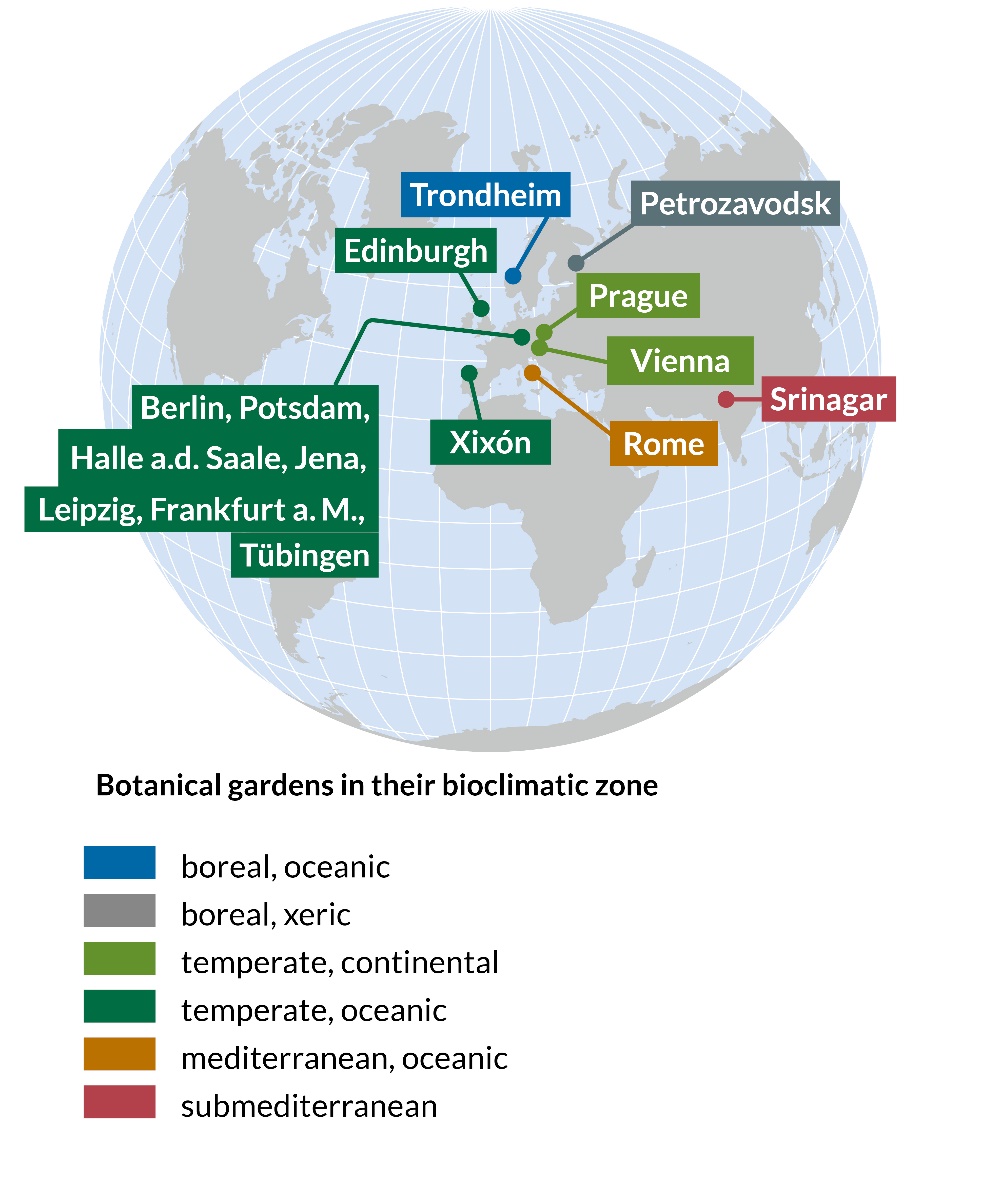


**Fig. 5** Map of all botanical gardens in which phenology of herbaceous species was monitored and used in this study


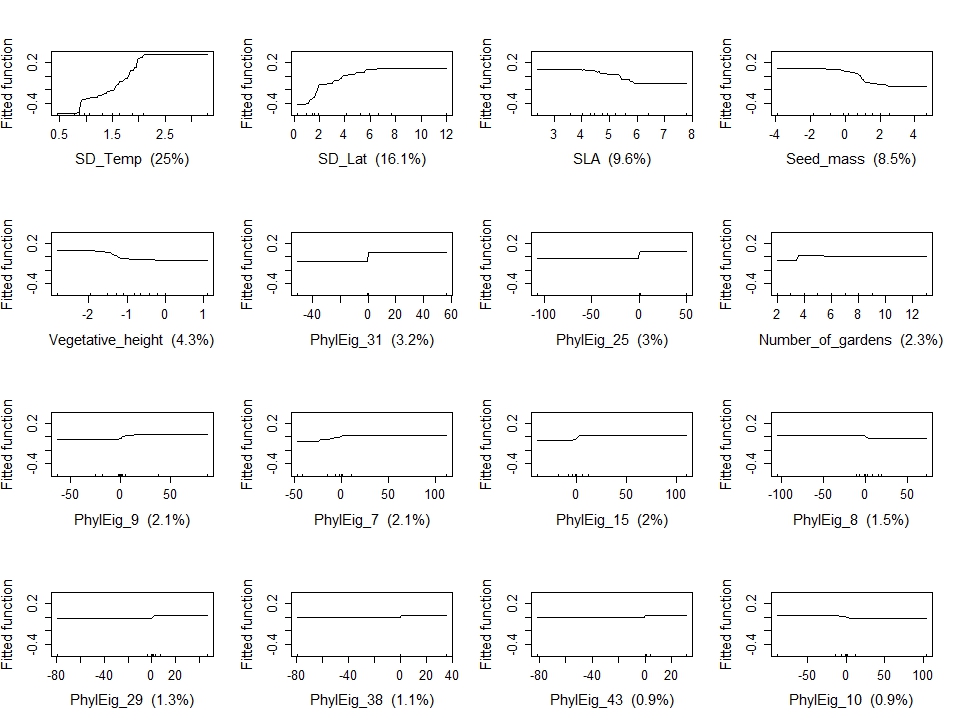


**Fig. 6A** Partial dependency plots of boosted regression trees for the relationship between ‘variability leaf unfolding’ and functional traits, variability in environmental conditions, phylogeny.


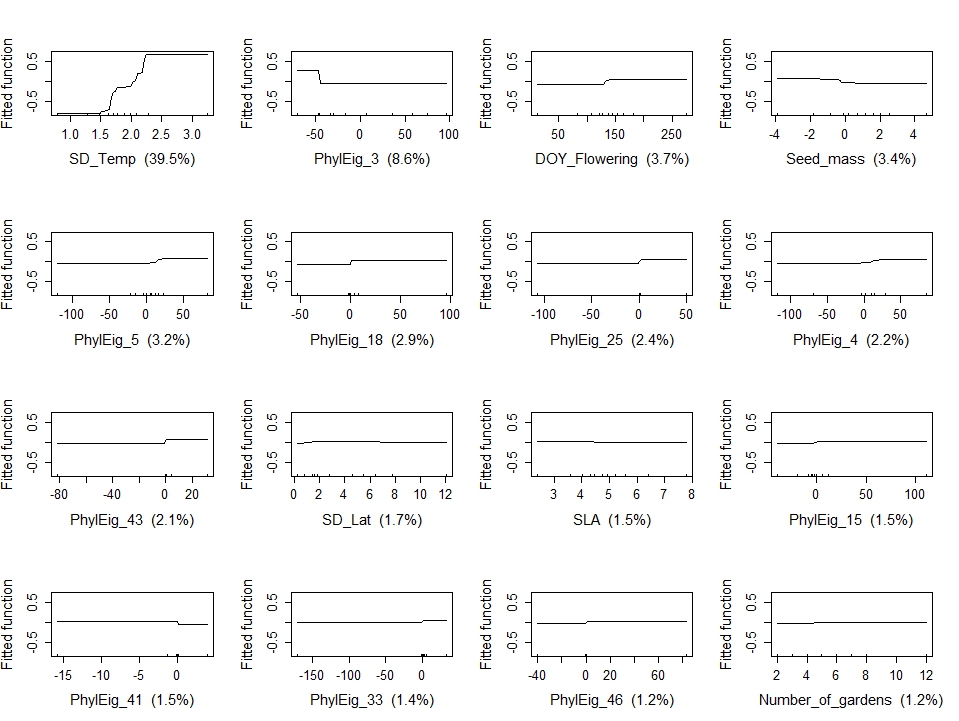


**Fig. 6B** Partial dependency plots of boosted regression trees for the relationship between ‘variability onset senescence’ and functional traits, variability in environmental conditions, phylogeny.


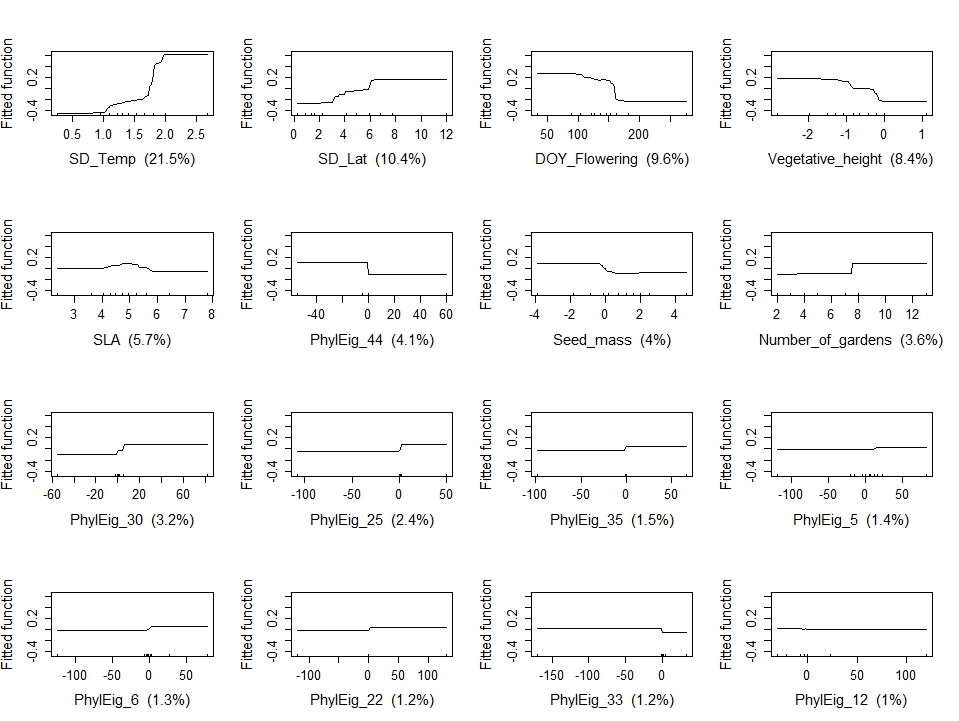


**Fig. 6C** Partial dependency plots of boosted regression trees for the relationship between ‘variability onset of flowering’ and functional traits, variability in environmental conditions, phylogeny.


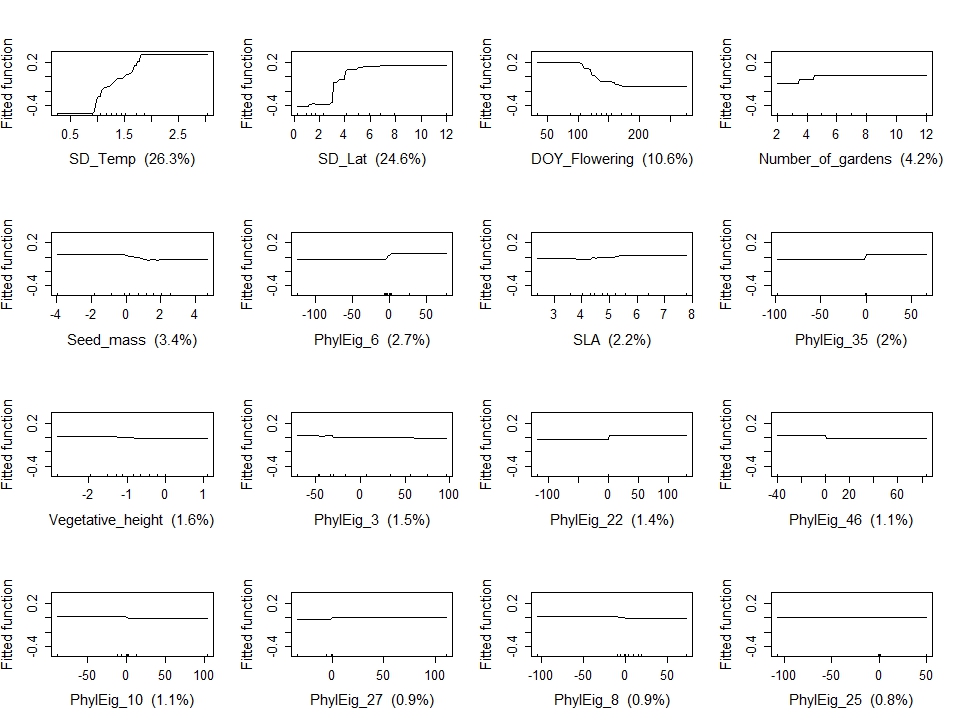


**Fig. 6D** Partial dependency plots of boosted regression trees for the relationship between ‘variability peak of flowering senescence’ and functional traits, variability in environmental conditions, phylogeny.


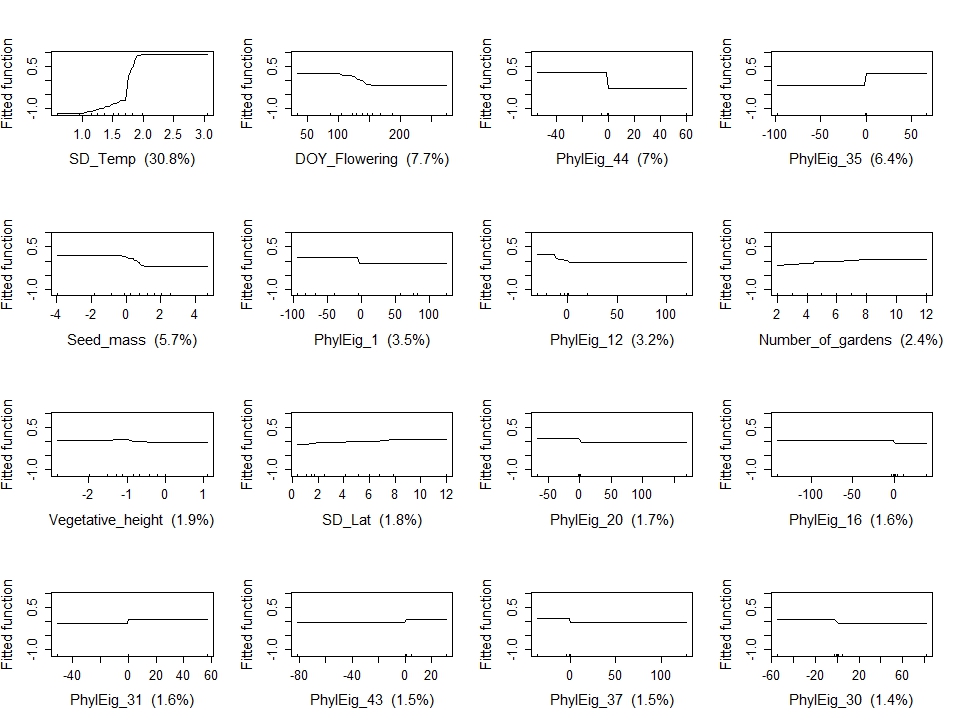


**Fig. 6E** Partial dependency plots of boosted regression trees for the relationship between ‘variability peak flowering duration’ and functional traits, variability in environmental conditions, phylogeny.


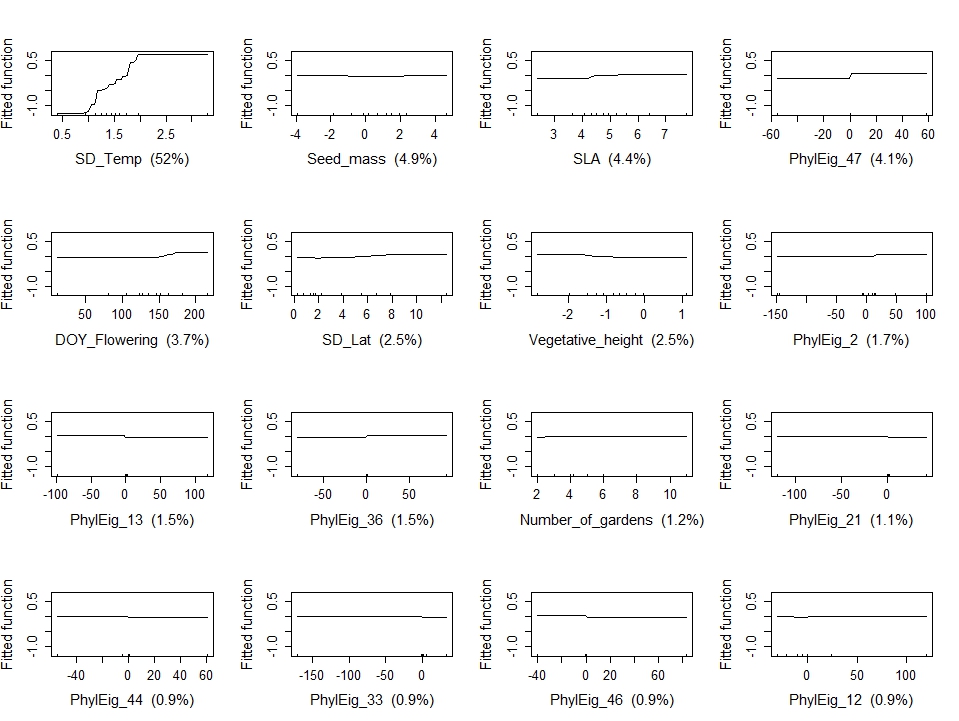


**Fig. 6F** Partial dependency plots of boosted regression trees for the relationship between ‘variability onset of fruiting’ and functional traits, variability in environmental conditions, phylogeny.
